# How an emergency department is organized to provide opioid-specific harm reduction and facilitators and barriers to harm reduction implementation: a systems perspective

**DOI:** 10.1186/s12954-023-00871-1

**Published:** 2023-09-21

**Authors:** Sunny Jiao, Vicky Bungay, Emily Jenkins, Marilou Gagnon

**Affiliations:** 1 School of Nursing, University of British Columbia, T201-2211 Wesbrook Mall, Vancouver, BC V6T 2B5 Canada; 2 School of Nursing, University of Victoria, 3800 Finnerty Road, HSD Building A402a, Victoria, BC V8P 5C2 Canada

**Keywords:** Harm reduction, Emergency department, Acute care, Unregulated substance use, Complex adaptive systems, Opioid agonist treatment, Supervised consumption, Withdrawal management

## Abstract

**Background:**

The intersection of dual public health emergencies—the COVID-19 pandemic and the drug toxicity crisis—has led to an urgent need for acute care based harm reduction for unregulated opioid use. Emergency Departments (EDs) as Complex Adaptive Systems (CASs) with multiple, interdependent, and interacting elements are suited to deliver such interventions. This paper examines how the ED is organized to provide harm reduction and identifies facilitators and barriers to implementation in light of interactions between system elements.

**Methods:**

Using a case study design, we conducted interviews with Emergency Physicians (*n* = 5), Emergency Nurses (*n* = 10), and clinical leaders (*n* = 5). Nine organizational policy documents were also collected. Interview data were analysed using a Reflexive Thematic Analysis approach. Policy documents were analysed using a predetermined coding structure pertaining to staffing roles and responsibilities and the interrelationships therein for the delivery of opioid-specific harm reduction in the ED. The theory of CAS informed data analysis.

**Results:**

An array of system agents, including substance use specialist providers and non-specialist providers, interacted in ways that enable the provision of harm reduction interventions in the ED, including opioid agonist treatment, supervised consumption, and withdrawal management. However, limited access to specialist providers, when coupled with specialist control, non-specialist reliance, and concerns related to safety, created tensions in the system that hinder harm reduction provision with resulting implications for the delivery of care.

**Conclusions:**

To advance harm reduction implementation, there is a need for substance use specialist services that are congruent with the 24 h a day service delivery model of the ED, and for organizational policies that are attentive to discourses of specialized practice, hierarchical relations of power, and the dynamic regulatory landscape. Implementation efforts that take into consideration these perspectives have the potential to reduce harms experienced by people who use unregulated opioids, not only through overdose prevention and improving access to safer opioid alternatives, but also through supporting people to complete their unique care journeys.

## Background

In April 2016, a public health emergency was declared in British Columbia, Canada in response to an alarming rise in substance related overdoses and deaths. These occurrences were attributable to a highly toxic drug supply characterized by unregulated opioids, such as fentanyl [1, 2]. In March 2020, a second public health emergency was declared in the province in response to the novel coronavirus (COVID-19) pandemic [3]. The pandemic further exacerbated the impact of the drug toxicity crisis and led to an increase in overdose events and deaths [4, 5]. The impact of the pandemic was palpable especially in its early stages. Whereas prior to the pandemic, people were discouraged from using alone as a safety measure, this was stymied by physical distancing measures implemented in response to COVID-19 [6]. In addition, the pandemic had an impact on people’s ability to access harm reduction services such as supervised consumption sites and withdrawal services [4, 5]. Most recently, between January and December 2021, there were 2,232 substance related deaths—the highest number ever recorded in a year, and representing a 26% increase from 2020, where fentanyl was detected in 86% of all substance related deaths [1]. The drug toxicity crisis and the compounding impact of the pandemic do not affect all people who use unregulated opioids equally. Social and systemic inequities create conditions by which people who live in poverty, Indigenous people, and people who are unstably housed are disproportionally affected [7–9].

Against the backdrop of dual public health emergencies, there is an urgent need for harm reduction interventions to be scaled up across all health and social care settings [10–15]. Harm reduction is a philosophy and a set of principles that inform policies, programs, and practices that aim to reduce the negative health, social, and legal impacts associated with drug use, drug policies, and drug laws [16, 17]. Harm reduction acknowledges that many harms associated with the use of unregulated substances—substances that are prohibited and criminalized in the Canadian context—can be attributed to systemic factors that marginalize, stigmatize, and oppress people who use substances including prohibition and criminalization [16]. Harm reduction began as a grassroots initiative led by people who use substances [18], and the approach has been taken up in health care settings to help decrease the harms related to unregulated opioid use. Examples of harm reduction in health care settings include the provision of naloxone kits, the distribution of safer injection supplies, supervised consumption services, drug checking, safer supply prescribing, and opioid agonist treatment [19–26]. These interventions have demonstrated benefits, including reducing infectious disease transmission, overdose deaths, substance use practices that lead to harms, as well as enhancing therapeutic relationships and increasing referral to services [27–30]. To date, many such interventions have occurred in community settings under the scope of public health and primary care [14, 26, 29].

There is currently a burgeoning of harm reduction interventions in the acute care setting, and in particular, interventions designed for people who use unregulated opioids. Harm reduction interventions are especially needed in this setting because people who use unregulated substances are more likely to rely on hospital-based services for their acute and chronic medical needs compared to the general population, and may resort to the hospital as a means to obtain primary care [31, 32]. In addition, the hospital has simultaneously been described as a “critical touchpoint” for people who use unregulated substances to access the health care system, as well as a “risk environment,” whereby factors such as abstinence-only policies and inadequate withdrawal management can intersect to contribute to an increased risk for substance use related harms [32, 33].

A number of acute care-based harm reduction interventions applicable to unregulated opioid use have been proposed and documented in the Canadian context, including making available addiction consultation services and inpatient supervised consumption services, providing opioid agonist treatment and take-home naloxone kits, distributing safer use supplies, as well as facilitating referrals to relevant community supports [32, 34–38]. Implementation of those harm reduction interventions in the context of emergency departments (EDs) hold particular promise. The ED is a source of both urgent and primary care for people who use unregulated substances, who often resort to the ED due to gaps in primary care and appropriate wraparound services in the community setting [39, 40]. In addition, an alarming 5.5% of individuals treated for non-fatal opioid overdose in EDs die within one year of their visit, of whom, 20.5% die within the first month [41]. The ED thus serves as a critical point of contact, offering opportunities to deliver potentially life-saving interventions, such as take-home naloxone kits, induction of opioid agonist treatment, and referrals for ongoing care [42, 43].

Available research on ED implementation of harm reduction interventions specific to people who use unregulated opioids is scarce. While many interventions are possible, the existing literature and clinical practice focuses predominantly on two interventions: take-home naloxone for people who have either experienced an opioid overdose, or are deemed as at risk for an overdose; and opioid agonist treatment, including treatment induction and referral to ongoing care in the community [20, 44–48]. Several studies address contextual factors that may influence ED implementation of harm reduction interventions, including factors at the service provider level, such as staff knowledge and attitudes [49–51], and factors at the policy level, such as policies for identifying eligible patients [49, 50]. Additional factors identified are at level of infrastructural support, such as having targeted electronic medical record systems alerts [49], and at the level of interdisciplinary/interagency engagement, such as having support from the hospital pharmacy, or mechanisms for transitioning to community-based services [52]. Overall, the existing literature tends to offer descriptions of how a *single* harm reduction intervention was developed, implemented, and evaluated, at the detriment of explorations into how the ED is organized as a *system* to provide harm reduction interventions. Furthermore, challenges to implementation are often attributed to small parts of the system that are deemed ‘broken’ [53], as opposed to articulating facilitators and barriers to implementation considering how these parts interact [54].

A systems perspective offers a unique and helpful lens to study harm reduction intervention implementation in the ED. There is a growing literature conceptualizing health care organizations, including EDs, as *systems* to acknowledge and appropriately attend to their multiple, interdependent, and interacting elements [54]. Consequently, interventions that aim to tackle challenges within health care organizations require an understanding of how the system is organized as a whole, and must also account for how various elements of the system interact with one another [54]. Conceptualizing the ED in this way allows for examination of how it is organized to provide harm reduction, while also enabling the identification of facilitators and barriers to implementation considering interactions between elements of the system. This type of understanding is urgently needed and can contribute to the formulation of tailored strategies—grounded in evidence—to better leverage opportunities for impactful implementation of harm reduction interventions in the ED.

### Purpose

Using the ED at a large hospital in Western Canada as a case study, the purpose of qualitative paper is to respond to this evidence gap. This paper has three objectives:

1. To describe the elements of the ED, as a system, involved in the organization and delivery of opioid-specific harm reduction
2. To describe the required interactions between various elements of the system to support harm reduction implementation
3. To describe the facilitators and barriers to harm reduction implementation associated with these interactions and their potential impact for the delivery of care

To our knowledge, there is no published research in the Canadian context that adopts a systems perspective to examine how the ED is organized to provide harm reduction interventions, nor identifies facilitators and barriers to implementation in light of interactions between system elements. Conducting such an analysis has implications for the development and implementation of ED-based harm reduction, with the potential to reduce the harms that are associated with unregulated opioid use.

### Theoretical framework

**Fig. 1:**
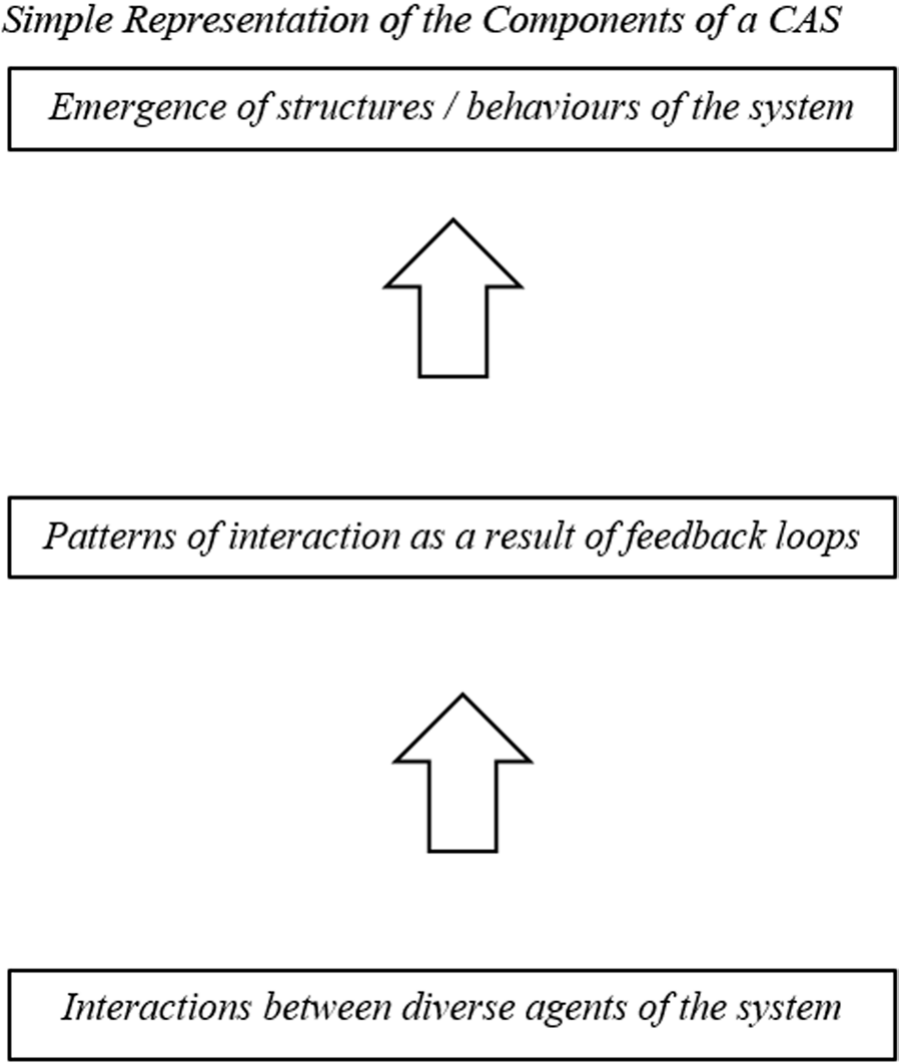
Simple Representation of the Components of a CAS. *Note*. Adapted from Evidence Scan: Complex Adaptive Systems by The Health Foundation [55], from https://www.health.org.uk/sites/default/files/ComplexAdaptiveSystems.pdf. Copyright 2010 by The Health Foundation

To address these research objectives, this study is guided by the theory of complex adaptive systems (CAS). A CAS approach considers systems, such as the ED, as a whole entity, and directs a focus on the emergence of system structures and behaviours as a function of patterns of interaction among system agents [54, 55]. Within a CAS framework, characteristics of a system are understood to arise from the characteristics of its agents and their interactions, yet they are not reducible to these characteristics [55]. In addition, one is unable to determine structures and behaviours of the system by observing the properties of constituent parts nor summing their behaviours [55]. Instead, a CAS has both positive and negative feedback loops, whereby the effect of any one agent’s activity can feedback on itself, as well as influence the activity of others [55]. The system engages in self-organization through the emergence of new structures and behaviours as a result of these feedback loops [55]. Thus, the characteristics that a CAS displays are not externally imposed, and are rather a function of patterns of interaction among its agents [55]. Although an agent’s range of interaction may be short, their range of influence is often wide [55]. Furthermore, interactions among system agents are nonlinear, where inputs are not proportional to outputs and small changes can lead to big effects. Although interactions among system agents often follow and are constrained by simple rules, complex behaviour can emerge in the system [55]. Please see Fig. 1 for an illustration of the components of a CAS.

The framing of complex adaptive systems has been utilized to study health care organizations in a number of ways, including to examine the roles and interrelationships within the healthcare workforce, to provide insight on how to manage health services, and to facilitate organizational development, change, and reform [56–64]. Most often however, CAS thinking has been applied to explore health service delivery, where this approach has been used to explore an array of topics, including clinical complexity, patient trajectories, clinical decision making, and health care teams [54, 65–70].

Although there has been previous work that has noted the need to study EDs as complex adaptive systems [54, 71, 72], there has been very limited empirical research that conceptualizes EDs as CAS. Of note, in a study by Nugus et al. [73], the authors examine how emergency clinicians manage dynamic boundaries between the ED and other hospital departments and organizations in striving to integrate care and meet the patient’s needs. Similarly, there has been a dearth of research that embraces EDs as complex adaptive systems in examining facilitators and barriers of implementing harm reduction interventions.

As research that adopts the lens of CAS has the potential to offer numerous benefits, including confronting dogged assumptions, delineating patterns of interactions between system agents, and gaining a more comprehensive understanding of forces that affect change [56], an analysis of how the ED is organized as a CAS to provide harm reduction interventions has the potential to contribute to the formulation of tailored, nuanced, and effective strategies to improve harm reduction implementation in the ED.

## Methods

### Research design

This qualitative paper draws on interview and policy data that were collected as part of a broader study known as *Harm Reduction in Emergency Departments (EDs) in [a province in Western Canada]*. This study utilized a case study design to examine how harm reduction interventions for unregulated opioid use are implemented in the context of EDs in a province in Western Canada, and to delineate the contextual factors that may influence harm reduction implementation. Case study methodology allows for an in-depth investigation into numerous dimensions of complex phenomena [74], such as those evident within complex systems like the ED. It is often used when the phenomenon of interest is too complex, context-bound, or context-sensitive to be studied in other ways [75]. Through drawing on various sources of data (e.g., interviews, surveys, text documents, etc.) to capture multiple facets of a phenomena [76], case study methodology allows for an examination of system elements at play, including the unique configuration of factors that serve as system facilitators or barriers, while also enabling those interrelationships to be considered in other similar systems. Case study methodology is compatible with the theoretical framework of CAS because, in both, systems are conceptualized as constituted by various elements. While systems theory is concerned with how interactions among system elements shape how systems self-organize, case study methodology is appropriate for examining systems as cases that have configurational arrangements that require parts to be understood in relation to one another and in terms of the coherent whole.

#### Setting

The case selected for this analysis is the ED at a large hospital located in Western Canada. The study site is an acute care hospital that serves a client population disproportionately impacted by health and social inequities, including people who use unregulated substances, people who experience homelessness, and people living with a diagnosis of HIV [77]. In 2015–2016, there were more than 500,000 visits to the hospital, comprising over 174,000 unique patients [78]. The hospital is known internationally for its expertise in harm reduction, including the development of a comprehensive set of harm reduction policies and the implementing of various harm reduction interventions. The hospital is also home to an in-house Addiction Medicine Consult Team, an onsite Rapid Access Addiction Clinic, and the second inpatient supervised consumption site in Canada [34, 79]. The hospital's ED opened in 1962 and is one of the busiest EDs in the province [80, 81]. In 2015–2016, there were more than 84,000 visits to the hospital's ED [78]. The ED serves a high prevalence of people who use unregulated substances, and is, in many ways, a pioneer in the Canadian context in the provision of care for substance use, offering interventions such as take-home naloxone kits and access to a low barrier to-go Suboxone® program [35, 36, 82].

Of all EDs that serve the local municipality, the study site's ED is the closest to a geographical area known as the Downtown Eastside (DTES). The DTES is an area where residents experience a multitude of intersecting systemic inequities including extreme poverty, precarious housing, interpersonal violence, intergenerational trauma, as well as ongoing racism and colonialism [83]. Substance use challenges are prevalent in the lives of many DTES residents [84]. In 2021, the highest rate of drug toxicity deaths in the province (49.5 per 100,000) was in the health authority region that encompasses the DTES [1]. Due to its geographical proximity to the DTES, the study site has played a critical role in responding to the drug toxicity crisis, with its ED seeing the highest number of substance-related overdose of any ED in the province, and almost 10 times more than other hospitals located within the health authority region that encompasses the DTES [85, 86].

### Data collection

Data collection included individual interviews and document review, which were collected concurrently.

We conducted a total of 20 in-depth interviews for this present study, with 15 of these being with ED nurses and physicians. Interviews used a semi-structured interview guide, and questions focused on the types of system agents that are involved in the delivery of opioid-specific harm reduction in the ED, how system agents interact to support harm reduction implementation, and facilitators and barriers to implementation in light of these interactions as well as their impact for care provision. Inclusion criteria were nurses (Registered Nurse and Registered Psychiatric Nurses) and physicians (staff physicians and resident physicians) who provide direct patient care. Interview recruitment occurred as part of the broader study during which time survey participants were asked to identify if they would be willing to participate in a subsequent interview. The 10 nurse participants all held Registered Nurse designations. Physician participants included four staff physicians and one resident physician. Although demographic data were not collected systematically, eight interview participants noted their length of work in the study site's ED. They reported various level of experience in the ED environment, ranging from 6 months to 12 years.

As data collection unfolded, it was apparent that clinical leadership was a critical aspect of harm reduction intervention planning and implementation. Thus, we conducted five additional interviews with key clinical leaders with responsibilities associated with harm reduction interventions at the broader level of the health authority. These interviews focused on their perspectives of policy, programming, and implementation of opioid-specific harm reduction interventions. Sampling of the clinical leaders was purposive, whereby participants were recruited for their experience in various roles, with the goal of capturing and understanding different angles and perspectives [87]. Clinical leaders who took part in the leadership interviews assumed various roles in the organization. For reasons of confidentiality, no details are provided as to their specific roles. All interview participants were offered a cash honorarium of $30 CAD. All interviews were audio recorded after obtaining consent from the participant.

Data collection also included the identification and review of text documents in the form of organizational policies (*n* = 9). Policies provide details pertaining to how the ED should function, encompassing information related to the roles, responsibilities, and availability of various system agents that are involved in the delivery of opioid-specific harm reduction. Policies also addressed how these agents are expected to interact with one another to support harm reduction implementation. Policies were gathered from the health authority's website. ED specific or organizational policies that addressed patients’ use of unregulated substances or harm reduction interventions were included. In total, nine organizational policies were gathered. (See Table 1 for policy title and purpose.).

**Table 1:** Included organizational policies and their purpose

Policy #	*Name*	*Purpose*
1	Harm reduction and managing substance use—Acute care [88]	To describe the organizational approach to managing substance use and implementing harm reduction interventions in the acute care setting
2	Philosophy of care for patients and residents who use substances [89]	To articulate a philosophy of care for people who use substances that aligns with the organization’s vision and mission
3	Violence prevention in the workplace [90]	To describe the organizational approach in the prevention of workplace violence, and to delineate the roles of relevant parties (e.g., leadership, staff, and occupational health and safety)
4	Injectable opioid agonist treatment (iOAT^1^) for opioid user disorder and IV fentanyl for withdrawal management [91]	To outline the protocols associated with the provision of iOAT and intravenous fentanyl
5	Dispensing take home naloxone kits to clients at risk of opioid overdose (adults and youth) [92]	To outline the protocol associated with the provision of take-home naloxone kits
6	Methadone for opioid use disorder [92]	To outline the protocol associated with the provision of methadone
7	Buprenorphine/naloxone (Suboxone®) for opioid use disorder [93]	To outline the protocol associated with the provision of Suboxone®
8	Buprenorphine/naloxone ‘(Suboxone®) to-go’ patient kits for induction outside of hospital setting [94]	To outline the protocol associated with the provision of to-go Suboxone® kits
9	Overdose Prevention Site (OPS) at [study site]: Operating procedures [95]	To outline operating procedures for the in-hospital OPS

Intravenous opioid agonist treatment (iOAT) involves replacing unregulated opioids with prescribed opioid medications in injectable form. Examples include hydromorphone and diacetylmorphine [91]

### Data analysis

Audio recorded interviews were transcribed and all identifying information was removed. Interview data were analysed using a reflexive thematic analysis (RTA) approach [96], and informed by the theory of complex adaptive systems [55]. As per the RTA approach, coding was an iterative process, where we did not employ a fixed codebook at the onset. Rather, codes evolved as more interviews were reviewed. In generating the codes used, we considered the types of system agents that are involved in opioid-specific harm reduction, how system agents interact to support implementation, and how patterns of interaction can lead to the emergence of systemic facilitators and barriers to implementation. As part of this process, a number of initial codes were deleted, renamed, split, and/or combined [97]. These refinements allowed for the gradual development of themes as meaning-based patterns and facilitated a fulsome interpretation of the data [97]. NVivo™ Version 12 was used in coding the interviews.

The theory of complex adaptive systems also informed the analysis of policy documents, which were reviewed to extract information pertaining to the roles, responsibilities, and availability of system agents involved in the delivery of opioid-specific harm reduction, as well as how these agents are expected to interact with one another to support harm reduction implementation.

To attend to triangulation, information extracted from policy documents was compared to the information provided by clinical leaders and ED staff to assess the extent to which the two were congruent, and whether there is alignment or disconnection. Both data sources contributed to descriptions of the types of system agents that are involved in the delivery of opioid-specific harm reduction, as well as how interactions between system agents can support harm reduction implementation. Interviews with clinical leaders and ED staff further enabled a nuanced analysis of how patterns of interaction among system agents led to the emergence of systemic facilitators and barriers to implementation, including the potential impact for the delivery of care. Considerations of how the two data sources interface with one another ultimately helped to shape the resulting analysis as well as the presentation of study findings.

## Results

**Fig. 2:**
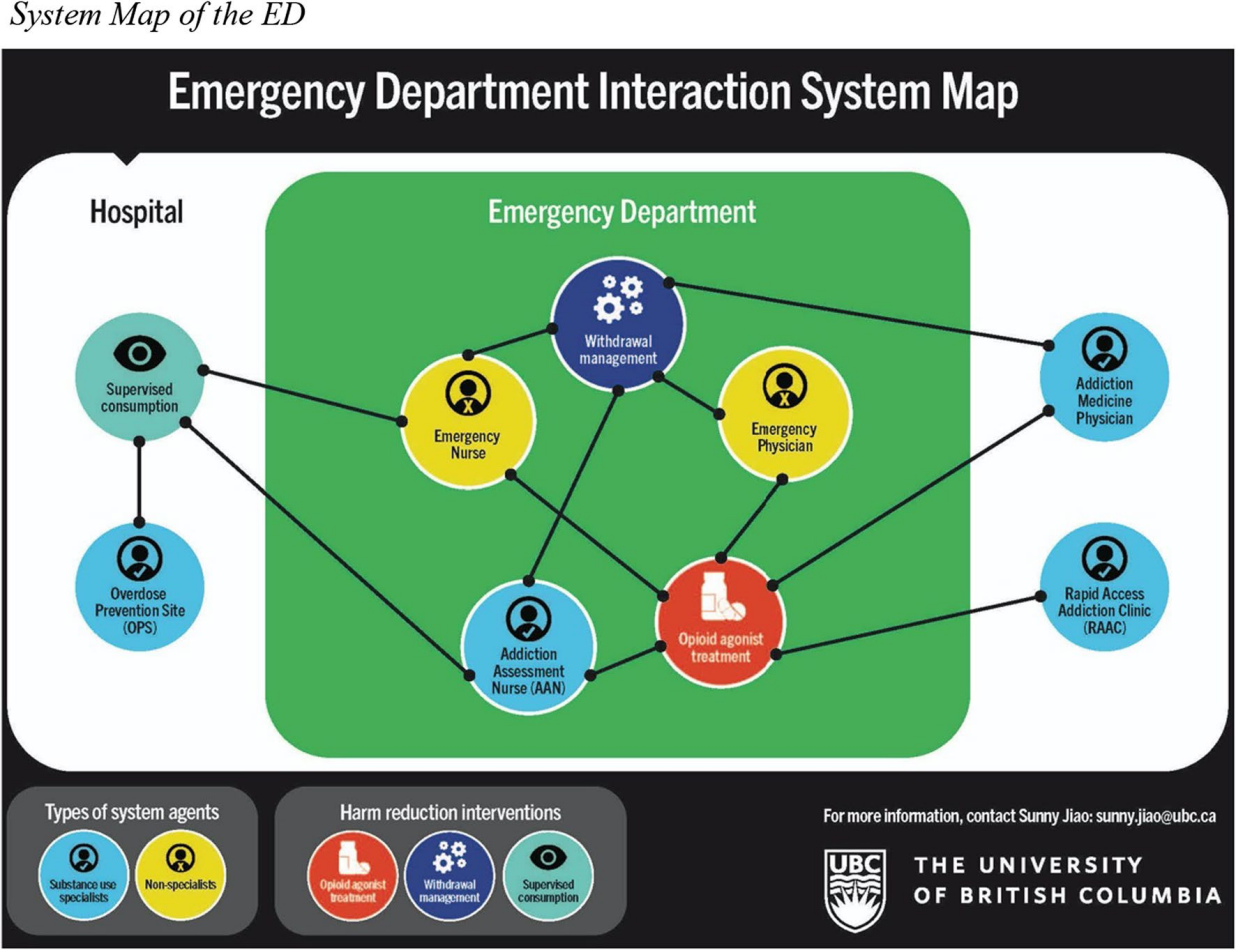
System Map of the ED. *Note*. This system map illustrates how various agents of the system are engaged within the context of different harm reduction interventions in the ED (OAT, withdrawal management, and supervised consumption). Its intention is not to capture the relationships between system agents

Our analysis showed that, in the context of the study site's ED, an array of system agents interact in ways that enable the provision of harm reduction interventions, including substance use specialist services (e.g., the Addiction Medicine Physician, the Addiction Assessment Nurse, and the Overdose Prevention Site) and non-specialist providers (e.g., the Emergency Nurse and the Emergency Physician). (See Table 2 for detailed descriptions of system agents and their roles. See Fig. 2 for a system map of the ED outlining the various interactions between system agents.). At the same time, limited *access* to specialized providers, when coupled with specialist *control*, non-specialist *reliance*, and concerns related to *safety*, creates tensions in the system that hinder harm reduction provision, where system agents are unable to work in synergistic ways to accommodate the 24 h a day service delivery model of the ED.

Following, we detail how *access* to specialist providers intersects with issues of *control*, *reliance*, and *safety* to influence the provision of three harm reduction interventions: opioid agonist treatment, supervised consumption, and withdrawal management, including the resulting implications for patients. While the types of harm reduction interventions and the factors influencing their provision are addressed separately for this analysis, we acknowledge the complex interplay between these interventions and influential factors, where one factor may influence the provision of multiple interventions, or the implications of one factor for the provision of one intervention may lead to the need for a second, different intervention.

**Table 2:** Diverse agents of the ED

System agent	Role
*Substance use specialist services*	
Addiction Medicine Physician	The Addiction Medicine Physician is responsible for assessing the patient concerning their unregulated substance use, including the severity of any presenting substance use disorders and stage of change, and establishing connections to treatment [97]. They also treat withdrawal, cravings, and pain with the goal of enabling the treatment of the patient’s admitting diagnosis [89, 97]. The Addiction Medicine Physician is available seven day a week, and are on-site from 8 am and 5 pm, and available by phone from 5 pm to 8 am [88].
Addiction Assessment Nurse (AAN)	Addiction Assessment Nurses (AANs) work in the ED and have expertise in care provision for people who use unregulated substances. They can assist with various aspects of care, including liaising with the Addiction Medicine Physician, obtaining information from community health providers, connecting patients to appropriate substance use related services in the community, helping to facilitate access to the in-hospital OPS, and providing mentorship and support to ED staff. They are available seven days a week from 8 am to 6 pm.
Rapid Access Addiction Clinic (RAAC)	The RAAC is an outpatient clinic that is located within the hospital that provides short-term treatment for people with substance use specific health concerns and connects people to care providers in the community for long term management [88]. The clinic facilitates connections to a variety of health and social services such as detoxification, counselling, housing, and financial aid [98]. It is open seven days a week from 9 am to 4 pm [88]. The clinic accepts referrals from the ED and inpatient units, community providers, and patient self-referrals [98, 99]. It has a mandate to see referred patients within a 24–48 h window, and walk-ins are seen on a first come, first served basis until capacity is reached for the day [99, 100].
Overdose Prevention Site (OPS)	Registered patients of the hospital, including ED patients, have access to the in-hospital OPS [88]. At the OPS, patients can use their personal supply of injection drugs and subsequently return to the ED to continue their care. The OPS is staffed by nurses who are responsible for overdose prevention and response, and harm reduction education and supply distribution, including take-home naloxone kits and safer use supplies [88]. The OPS is open from 10 am to 8 pm seven days a week [88, 95].
*Non-specialist providers*	
Emergency Nurse	The Emergency Nurse's role is expansive. For the purpose of this paper, which pertains to care provision for people who use unregulated substances, the Emergency Nurse is responsible for ensuring that the patient’s substance use related needs are met through advocating for any necessary interventions on their behalf, providing treatments/interventions ordered, assessing effectiveness, providing patient education, and helping to coordinate all aspects of care provision related to substance use, both within the ED and through substance use specialist services that exist outside of the ED environment.
Emergency Physician	The Emergency Physician's role is expansive. For the purpose of this paper, which pertains to care provision for people who use unregulated substances, the Emergency Physician is responsible for ensuring that the patient’s admitting diagnosis is attended to, and that any needs related to substance use are also addressed, either through their own efforts or through referral to substance use specialist services, such as the Addiction Medicine Physician, the Addiction Assessment Nurse (AAN), and the Rapid Access Addiction Clinic (RAAC).

### Opioid agonist treatment: the interplay of access and control

For some who use unregulated opioids, their goal may be to access opioid agonist treatment (OAT), which works by replacing an unregulated opioid with prescribed opioid medications. OAT is a harm reduction intervention that can help to prevent withdrawal and reduce cravings. It can also offer a safer alternative to unregulated opioid use, which is vital in light of the ongoing drug toxicity crisis [101].^1^ The study site's ED offers a number of options for OAT, including diacetylmorphine and hydromorphone as iOAT, and methadone, Suboxone® [91, 93–95], and Kadian® as oral OAT.

An array of system agents, including both substance use specialists and non-specialists, interact with one another to enable the provision of OAT, as illustrated in Fig. 2. Of which, the Addiction Medicine Physician—a substance use specialist—plays a most critical role. If a patient presents to the ED and is interested in OAT, the Emergency Physician can make a referral to the Addiction Medicine Physician, who can assess the patient and order the most appropriate form of treatment [91, 93–95].^2^ A referral can also be made to the Addictions Assessment Nurse, who can assess the patient and subsequently liaise with the Addiction Medicine Physician to obtain orders for OAT.

While the Addiction Medicine Physician as a specialist service *enables* the provision of OAT, it simultaneously *controls* provision. This is because, for most types of OAT, with the exception of Suboxone®, the involvement of the Addiction Medicine Physician is mandatory—either the Addiction Medicine Physician has to order the treatment, or the Emergency Physician needs to consult with the Addiction Medicine Physician prior to ordering OAT [91, 93–95].

Pharmacy, I don’t think will release Kadian [slow release oral morphine] to us unless we speak to the Addictions Physician on call. […] Last time I tried to do it, there was that extra piece, and I think methadone as well had the same issue. So when I tried to give methadone for a patient who required their evening dose, the pharmacy refused to release it without a conversation with Addictions.—ED staff

Despite the extent of *control* that the Addiction Medicine Physician has over the provision of OAT, there are several limitations on *access* to this specialist service—in terms of *which types* of ED staff can make the specialist referral, *when* different types of staff can access the specialist service, and when it would be *appropriate* to contact the service afterhours.

First, referrals must come from the Most Responsible Physician (the medical service under which the patient is admitted in the hospital, which in the ED setting, is often the Emergency Physician), the Addiction Assessment Nurse, or the Clinical Nurse Leader [88]. That is, the Emergency Nurse, who has the most direct contact and engagement with the patient is not able to make such a referral and must go through other care providers.

We [Emergency Nurses] can’t call Addictions, the MRP [Most Responsible Physician] has to be the person that calls Addictions. So we don’t call Addictions. That’s just what is. That is directed from our on-call list, it’s just not allowed for us to call. If it was the ERP (Emergency Physician) or if they were admitted under CTU or GI [other medical services under which a patient can be admitted], those people would have to call.—ED staff

Second, once a patient has been referred to the Addiction Medicine Physician, the Emergency Nurse has direct access to (and can call) the Addiction Medicine Physician only during the daytime hours of 8 am to 5 pm. Only the Emergency Physician has direct access to the Addiction Medicine Physician outside of these hours [88].

Third, there are limitations on when it would be appropriate to contact the Addiction Medicine Physician afterhours. The Emergency Physician can contact the specialist service for guidance on new patients, or “when the clinical team has attempted all available strategies and substance use issues are still unmanaged” [88] (p. 6). The Emergency Nurse can advocate for such contact for issues including but not limited to: uncontrolled opioid withdrawal, and order clarification for medications ordered by the specialist service (“try to call before 2200 [10 pm]” and “ideally for orders that cannot wait until the following morning to be clarified”) [88] (p. 6). Policy dictates that the Emergency Nurse is also not to advocate for contact for pain management, with the stated assumption in the policy that pain may indicate an acute medical issue. Instead, if the patient is experiencing new onset or worsening pain, they are to ask the Emergency Physician to assess, who can then address the pain or contact the afterhours Addiction Medicine Physician if they deem there is a need to do so [88].

It is perhaps, in part, due to these various limitations that there is a great discrepancy in how staff in the ED speak about afterhours access to the Addiction Medicine Physician. While some ED staff are certain that this specialist service is available 24 h a day, others have voiced that this has not been their experience. That is, although *in theory* the Addiction Medicine Physician is available around the clock, this may not be the case *in practice*. These discrepancies are highlighted through participants who report conflicting experiences in practice. For example, one participant shared:

Addictions is always available by phone. […] If the patient requires Kadian at eight o’clock at night, we would call them, they’d call back, we’d get approval of the Kadian® and the Kadian® comes up from pharmacy and the patient gets their drug.—ED staff

And yet, another participant had quite a different experience when trying to access the Addiction Medicine Physician, stating:

The Addictions team [is] not [available] around the clock. Our Addictions Nurse is there until maybe like five or six, and sometimes the Addictions Medicine team is around that time as well, but sometimes the plan of care of patients in our department [is] "Addictions to see in the morning” or “Addictions Nurse to see in the morning,” so overnight they are not accessible.—ED staff

The interplay between specialist *control* over OAT provision and *access* to these same specialists can create tensions in the system that hinder ED staff ability to comprehensively and consistently provide this specific harm reduction service to patients. If, for instance, a patient attends the ED after 5 pm and is interested in OAT, they will not be able to see the Addiction Medicine Physician. Instead, at the point of discharge, they will be referred to an outpatient service, such as the in-house Rapid Access Addiction Clinic (RAAC), to initiate OAT. However, the RAAC is also not available at all hours of the day (9 am to 4 pm). After the RAAC is closed, the Emergency Nurse can provide the patient with a brochure about the clinic.

The provision of a brochure to people who are simultaneously navigating competing life priorities, such as securing food or shelter, or managing withdrawal, is insufficient. This is because these competing needs render the patient less likely to return to attend the RAAC on a different day. ED staff members speak to how the patient’s visit to the ED is a window of opportunity to facilitate access to OAT, and how due to limited access to specialist services such as the Addiction Medicine Physician and the RAAC, these opportunities are missed:

What is really unfortunate is people that come in the middle of the night […]. There is no Addictions doc and RAAC is not open. […] We can tell them to go in the morning but they never do because there is a very small window.—ED staff

If RAAC is closed, which is two-thirds of the time at least, you would say “Here is a pamphlet and I encourage you to go to RAAC in the morning.” That obviously is just so much less of a closed loop. I think the chances of us getting them is just so much lower, but I do that all the time because that is just our reality.—ED staff

#### Withdrawal management: the interplay of access and reliance

People who use unregulated opioids are at significant risk for withdrawal while accessing care due to factors such as prolonged wait times, abstinence-based policies, and the inadequate recognition and management of withdrawal needs [33, 102–104]. Untreated opioid withdrawal is extremely uncomfortable and distressing for the patient, and is associated with the use of unregulated opioids and a high risk of overdose deaths after discharge from the ED [105]. There is further evidence that unmanaged withdrawal can lead to the patient leaving without having their care completed [33, 106, 107].

Withdrawal management is a harm reduction intervention available in the study site's ED (Fig. 2). When people attend the ED and are undergoing opioid withdrawal, or are at risk for withdrawal, the Emergency Physician has the authority to order the treatments required, which is typically an opioid medication, such as hydromorphone or morphine. However, as a non-specialist provider, the Emergency Physician may not feel that they have the expertise required, or they may not be comfortable ordering the doses needed, as conveyed by study participants below:

So sometimes the dosing is unclear, so if somebody is on 1000 [mg] of Kadian a day, it is difficult for, because the amounts are way out of what we’re used to, what we’re comfortable with in terms of dosing of narcotics, and so when we’re giving and trying to translate into how much Dilaudid does this patient need to stop their acute withdrawal.—ED staff

I have a ton of experience with this patient population but I would still say that I probably don’t give sufficient doses. […] I wouldn’t say I’m not comfortable with, I’m not worried about that I’m going to overdose these patients. […] It’s just that I perhaps don’t appreciate what frequency of dosing and how large the dosing sometimes needs to be. So I’m certainly happy to make an effort, but I would bet that I often undershoot.—ED staff

Participants also spoke to how the patient’s clinical complexity related to active opioid use, acute illness, and the ever-changing drug supply, can further complicate withdrawal management:

I would say the most worrying part is when you’re giving those high doses and the patient is using as well, just because then you have no real gauge of how much they’ve actually had. What is harder is […] when the patient is really sick and you’re trying to also handle their withdrawal needs, so it’s hard to tell the clinical picture from that, they’re delirious and tachycardic, restless, agitated, but drowsy, and it’s hard to gauge if you should be giving more or less opioids.—ED staff

I couldn’t have even fathomed that there would be an additional thing [benzodiazepines] that would be in the drug supply to make life harder. […] So it is an added factor, […] what we can give you to meet your needs in respect to your withdrawal and your pain, if you just used a whole bunch of fentanyl, which is what you’re used to, but unknowingly you’ve also used a bunch of benzodiazepines. […] It makes it a lot harder if you don’t know exactly what is on board.—ED staff

Consequently, the Emergency Physician may need to *rely* on their specialist colleagues for withdrawal management, which can be facilitated through interactions with several types of specialist providers, as presented in Fig. 2. Below, an ED staff member speaks to the option of referring to the Addiction Medicine Physician, who can assess the patient and order the treatments required:

I think lots of people [Emergency Physicians] will call the Addictions team [to assist with withdrawal management] and some people are comfortable to give what tends to be an arbitrary dose [of medication for withdrawal management].—ED staff

The Emergency Physician can also refer to the Addiction Assessment Nurse, who can assess the patient and share results of their assessment with the Addiction Medicine Physician, who can order the required treatments. Alternatively, the Addictions Assessment Nurse can make a treatment recommendation to the Emergency Physician, as shared by the following participant:

They’re [the Addiction Assessment Nurses] very attuned to figuring out what the patient’s history is, what’s appropriate for this patient, their dosing levels based on what they’re getting prescribed on paper but also what their substance use is in real life. And then helping to advocate for that. […] The AANs are recognized as experts in this area, and so they advocate and they make a recommendation to say, the Emergency physician, that is often received very well and they trust that advice, and they’ll actually usually act on it.—Clinical leader

Although the Emergency Physician may *rely* on specialist providers in managing opioid withdrawal, as noted previously, the Addiction Medicine Physician is, in effect, available from 8 am to 5 pm. Additionally, the Addiction Assessment Nurse is available from 8 am to 6 pm.

The reliance of non-specialist providers on specialist services, when coupled with limited access to these same services, can translate to increased patient suffering and distress as a result of the patient’s withdrawal needs not being adequately met, which was the experience of the participant below:

I always find it very helpful if it’s daytime, it’s 10 am, and I call Addictions and they come down. […] It is incredibly helpful to have that support. Afterhours, that is really tricky and I’m sure those patients get suboptimal care.—ED staff

While during the day, the Addictions Assessment Nurse may be able to advise the Emergency Physician as to what would be an appropriate dose for withdrawal management, the Emergency Nurse may not have the same expertise to do so afterhours:

If an Emergency Nurse tries to advocate for, “We need to something for their withdrawal,” and the doctor says, “Well, what do you want to give them?” “Well I don’t know. I don’t know what they need. That’s not my area of expertise.”—Clinical leader

Implications for the patient are substantial, include using at the bedside, in the patient bathrooms, or using outside, thereby posing a risk for unwitnessed overdose. Patients may also be forced to leave without completing the necessary care for the urgent health issue that necessitated their ED visit. An ED staff member speaks to the implications of not having access to the Addiction Medicine Physician afterhours:

If it’s afterhours, they don’t have Addictions to call, so I think it gets much less, done much more poorly then. I think what ends up happening is people leaving against medical advice [without having their care completed] because they’re in withdrawal and they’re not getting that, and that’s a big problem. I think that is a very, very real phenomenon. Very common.—ED staff

#### Supervised consumption: interplay of access and safety

The use of unregulated substances by patients, including opioids, is not permitted in the ED and policy states that this rule is due to safety concerns related to unwitnessed overdose [88], which, as noted earlier, is amplified in the current toxic drug supply crisis, especially related to the use of unregulated opioids such as fentanyl [1]. However, given patients' expertise in what substances are most appropriate for them [108], they may wish or need to use their own unregulated opioids while attending to care.

The ED acknowledges the reality of these circumstances and consequently provides a harm reduction intervention, the Overdose Prevention Site (OPS), to enable in-hospital substance use (Fig. 2). The OPS is a safe space to inject substances under the supervision of nurses who are able to respond in the event of an emergency [88, 109]. A clinical leader speaks to the role of the OPS to prevent unwitnessed overdose and promote safety:

Keep our patients safe, give them a safe place to go and use, […] so that patients aren’t using at their bedsides, patients aren’t using in stairwells and nooks and crannies, and overdosing.—Clinical leader

A number of system agents are involved in facilitating access to the OPS (Fig. 2). ED staff are to let patients know about the availability of the OPS and patients can attend the OPS as long as their clinical condition allows them to do so. A clinical leader elaborates on the approach taken by ED staff:

Respectfully telling them [the patient], “you’re not to use in the bathroom, in the bed, it’s not safe due to risk of overdose, it’s just not safe.” So, we direct patients to the OPS if they are able to go there.—Clinical leader

The Addiction Assessment Nurse (AAN) can help ED staff make decisions about whether a patient is clinically appropriate to attend the OPS. A participant speaks to this aspect of the Addiction Assessment Nurse role:

The AAN can use their clinical judgement on whether or not someone should go up to the OPS at that time. Sometimes there are other sort of interventions that we can use to keep them in the department and depending on how much they need to stay there versus how safe it is for them to leave. It is really important to have that person with the clinical knowledge to support those decisions.—Clinical leader

In addition, as the patient’s absence from the ED while attending the OPS may impact care provision, the Addictions Assessment Nurse can liaise with OPS staff to provide an estimated time for the patient’s return to the ED to resume care. The role of the Addictions Assessment Nurse as a liaison between the ED and the OPS is noted by a clinical leader:

Any time that there are patients in ED who are looking to use substances, the AAN is sort of that bridge to getting someone from ED up to the OPS. They’re able to liaise with both teams and that way, if somebody is leaving ED, the staff in ED know that that patient is looked after, they know that they’re gone to the OPS, they know there is somebody they can call with an ETA on when that person is coming back.—Clinical leader

Although the OPS may be the only option for patients who wish to use opioids in a safe manner while they are accessing care, this specialist service is only available from 8 am to 6 pm. An ED staff member speaks to their experience of having limited access to the OPS:

Unfortunately, the Overdose Prevention Site is not as accessible as it was, it was never open 24 h so everything that happens at night, you’re going to be dealing with that.—ED staff

The interplay between concerns related to *safety* and limited *access* to a specialist service that is meant to serve as a workaround solution has profound implications for the patient. When the OPS is not available, the patient may defy organizational policy and use in the immediate ED environment. They often resort to using in isolated areas, such as at the bedside or in the patient bathrooms in order to avoid detection by ED staff, posing a risk for unwitnessed overdose.

Although the use of unregulated substances by patients, is not permitted, if a patient was found to be using in the immediate ED environment, they are not automatically discharged, or denied access to care. Instead, ED staff are to assess for safety and respectfully remind the patient that they are expected not to use in the department as it is unsafe [88]. They are to let the patient know about the availability of the in-hospital OPS. They are also to inform the Addiction Medicine Physician about the in-hospital substance use to see about ways that the health care team can work with the patient to better manage their needs, such as through offering medications for withdrawal management, or options for OAT [88], although the availability of these options may be affected by issues related to *control* and *reliance* as noted previously.

Despite the options available, however, the patient may not be interested. Prohibited from their own unregulated opioids, some patients may leave the ED and ultimately not receive the care that they need. Below, an ED staff member and a clinical leader speak to employing various strategies to support patients to complete care, and how this is not always possible.

I straight away offer anything that’s already ordered PRN, to help with the withdrawal symptoms or pain, and then if we have already given as much as we have been able to, I’ll say “I can call the Addictions team and see if we can get you something more,” and sometimes that might work for a short amount of time, but if they are very adamant, we can’t hold them. We can either say there is a safe injection site, because I always try to get out of them why they want to leave and what is their reasoning. Sometimes I ask straight up, “Do you want to use?” […] Just trying to understand exactly what their needs are and try to meet them, which you can’t always do.—ED staff

Other patients may continue the use of unregulated substances in the ED environment. If substance use is ongoing, especially when there are "unsafe behaviours" that may jeopardize the safety of others, such as open flames, uncapped needles, unregulated substances left attended, or verbal or physical aggression [88], the health care team may initiate a Behavioural Support Plan with the patient, which conveys that if the patient does not stop the behaviour in question, they may be forcibly discharged and lose their bed [90]. Similar to aforementioned barriers to harm reduction provision, this scenario also decreases the likelihood of the patient receiving care. A clinical leader speaks to how the ED manages unregulated substance use that is deemed unsafe—an approach that is also reflected through organizational policy [89, 90].

If substance use is ongoing, especially if it’s in a unsafe manner, then we’ll start to think about a care plan for the patient and if the behaviour is unsafe, then we can look at what’s called a Behaviour Support Plan […], it will say “This is the unsafe behaviour,” it’s almost like a contract with the patient, “Here is what is unsafe, here is what we’ll do instead” and then “If I don’t stop doing this unsafe behaviour then I might be discharged,” sometimes that’s the outcome.—Clinical leader

## Discussion

Using the ED at a large hospital in Western Canada as a case study, this paper examines, through a systems lens, how an ED is organized to provide harm reduction interventions in an unregulated opioid use context, and identifies facilitators and barriers to harm reduction provision in light of interactions among various agents and elements of the system.

Study findings demonstrate that diverse system agents, including substance use specialist services and non-specialist services, interact with one another to enable the provision of a number of harm reduction interventions, including opioid agonist treatment, supervised consumption, and withdrawal management. Existing studies report on the benefit of having substance use specialist services available in the acute care setting in enabling an array of harm reduction interventions including withdrawal management, safe opioid prescribing, naloxone prescription, the induction of opioid agonist treatment [110–112]. However, these studies do not adopt a systems perspective, nor address how specialist services facilitate harm reduction provision in light of interactions with other system agents. To assess the role of a system agent in contributing to system success or failure without considering its various interrelationships can lead to the formulation of strategies that are ineffective and produce unintended consequences or suboptimal outcomes [53, 54].

Findings also highlight the resounding impacts of limited access to substance use specialist services, especially the Addiction Medicine Physician and the Overdose Prevention Site. Although the ED has the potential to make harm reduction interventions such as OAT induction and withdrawal management available at all hours of the day, limited access to substance use specialist services can mean that this potential varies throughout the day, and the system is unable to support harm reduction provision in a way that is consistent with the around-the-clock service delivery model that is associated with the ED. Previous literature that describe substance use specialist services that operate in the acute care setting have similarly reported that access to these services is limited, especially on the weekend, where care providers have voiced frustration that patients who were admitted or discharged over the weekend were not receiving life-saving substance use related services [111]. Even among specialist services that were available to provide in-person consults on the weekend, this was tasked to trainees [111]. Given the immense benefits of having access to substance use specialist services, availability that is not congruent with operating hours for settings like the ED is problematic. There is little reason that, while the ED care delivery model recognizes that people's health care needs occur at all hours of the day, the same recognition does not apply to serious, substance use related needs, such as withdrawal management and overdose prevention. This concern is significant, especially in light of evidence that more than half of referrals to acute care based substance use specialist services originate from the ED [79].

While the existing research only point to a lack of staffing resources as a reason for limited access to addiction consultation services [111], our study draws attention to the impact of organizational policy on access—policies that prohibit the Emergency Nurse from advocating for contact with the afterhours Addiction Medicine Physician for concerns related to pain management, and from having direct access to the Addiction Medicine Physician afterhours. Unspoken in these restrictions is the assumption that nurses are unable to assess patients independently or appropriately—an assumption that is not supported by the scope of practice for nurses in the province [113]. These restrictions speak to hierarchical relations of power as applied to nurses and physicians in determining patient needs, and consequent to such restrictions, nurses are underutilized and unable to practice to their full professional scope, contributing to missed opportunities for harm reduction provision. Aside from explicit restrictions imposed by organizational policies, there are also implicit restrictions, as evidenced by a discrepancy between the Addiction Medicine Physician's hours of availability in theory versus in practice. Findings in this paper illustrate the need for a more nuanced understanding of what is shaping such discrepancies, and how ED staff are experiencing and navigating them.

Findings also underscore the impact of specialist *control* on the provision of harm reduction, where, with the exception of Suboxone®, all types of OAT require the involvement of the Addiction Medicine Physician. Previously to 2018, physicians in Canada were required to obtain an exemption under Sect. 56(1) of the *Controlled Drugs and Substances Act* prior to prescribing or providing methadone. As a response to the drug toxicity crisis, and in an effort to increase access to OAT, this requirement was removed in May 2018 [114]. Furthermore, a Provincial Opioid Addiction Treatment Support Program (POATSP) has been available to all physicians in the province since 2017, which, upon program completion, allows physicians to prescribe the full range of OAT and iOAT options, including methadone, Suboxone®, Kadian®, and injectable hydromorphone and diacetylmorphine [115]. While these changes relay the message that harm reduction should not be limited to the practice of specialists, this perspective has not been reflected in hospital policies, potentially jeopardizing the capacity of the ED to provide OAT as a harm reduction intervention. While it would be naïve to assume that organizational policy is the only barrier to non-specialist prescription and provision of OAT, there is nevertheless the need to re-examine the possibility of policy changes in keeping with regulation reform (prescribing), while, at the same time, exploring additional barriers, such as a lack of education or training, or concerns related to patient safety.

In the system that is the study site's ED, multiple barriers to harm reduction implementation relate to restrictions placed on the Emergency Physician's scope of practice, such as those pertaining to the ordering of OAT, thereby hindering the ability of the Emergency Physician to provide the full scope of harm reduction interventions. It is clear that there is prevailing, hegemonic discourse that care provision for people who use unregulated substances is a highly specialized practice—a discourse that is also reflected through how organizational policies were constructed. Harm reduction, however, began as a grassroots movement instigated by people who use unregulated substances and their allies [18], and was never intended to be constrained to the realm of experts. Yet, harm reduction has been co-opted into the biomedical paradigm, which privileges the knowledge of "experts" and the need for specialization [116]. It is worth considering why the ED is reinforcing the need for expertise and specialization when this type of discourse is impeding harm reduction provision. There is also a need to inquire into how Emergency Physicians are experiencing, and taking up policies, that restrict their scope of practice, and how they situate themselves in the conceptual tension of harm reduction as a technical solution versus a contextualized social practice [116].

Finally, study findings underscore that appropriate harm reduction interventions not only helps to prevent unwitnessed overdose, and increase access to safer opioid alternatives, but can ultimately supports patients to complete their care in the ED. There is an abundance of evidence that people who use unregulated substances may leave the acute care setting without completing care due to factors such as unmanaged withdrawal and ongoing cravings to use substances [33, 103, 107, 117, 118]. By making available harm reduction interventions, such as supervised consumption and withdrawal management, the ED can attend to these needs and support patients to complete care. Conversely, ongoing substance use in the immediate ED environment, especially if there are related safety concerns, can jeopardize care completion, whereby the patient may be asked to participate in behaviour contracting, which may eventually lead to forcible discharge. Previous research in the Canadian context has similarly reported on the use of behavioural contracting and threats of/actual discharge to enforce expectations of abstinence in the acute care setting [104]. These authors found that, due to a lack of institutional policy, care providers took vastly different approaches when they encountered active substance use, including "turning a blind eye," providing a safer opioid alternative, discharging the patient, and encouraging the patient to use outside and return [104]. In contrast, our study found that care providers made earnest attempts to help the patient meet needs related to substance use, such as though offering OAT, managing opioid withdrawal, and inviting the patient to attend/facilitating access to the OPS, prior to resorting to the use of behavioural contracting, or forcible discharge.

### Limitations

There are several limitations to this study. First, due to the self-selection of qualitative interview participants, it is possible that study findings represent the insights and experiences of ED staff who are more accepting of harm reduction as a practice, and more willing to engage in harm reduction implementation. Second, in alignment with our theoretical framework of complex adaptive systems, we tried to capture the perspectives of various system agents, including Emergency Nurses, Emergency Physicians, and clinical leaders. We did not include occupational groups such as social workers or security personnel who work in the ED given that Emergency Nurses, Physicians, and clinical leaders are most directly involved in the implementation of harm reduction in the acute care context. The strength of this approach is that we were able to attain a very nuanced understanding of the perspectives of those who are in a position to implement, or affect the implementation of harm reduction. However, we were unable to include Addiction Medicine Physicians in our study sample. There is a need for a larger scale examination of harm reduction implementation in the ED that considers the perspectives and experiences of all stakeholders, including patient perspectives. Third, despite the use of case study methodology, which supports the generation of highly contextual findings, we believe that our study findings hold relevance for other EDs in the province and across the country, regardless of their current stage of harm reduction implementation. For EDs that have harm reduction interventions that are underway, study findings can provide opportunities for further optimization through illustrating the need and importance of attending to interrelationships between system agents. For EDs that have limited engagement in harm reduction provision to date, but are looking into the possibility of future implementation, study findings can similarly provide valuable guidance.

## Conclusion

In the advent of an ongoing drug toxicity crisis related to the use of unregulated opioids in Canada, there is an urgent need to expand harm reduction approaches across all service provision settings, especially in the ED, which serves as a critical point of connection for people who use unregulated substances to the health care system. Existing research on harm reduction implementation in the ED context is scarce, and often offers descriptions of how a single harm reduction interventions was developed, implemented, or evaluated. In contrast, the present study delves into how the ED is organized as a *system* to provide harm reduction interventions, and identifies facilitators and barriers to implementation in light of interactions between diverse system agents, while considering the interplay between access, control, reliance, and safety. The findings of this study are novel and unique, and have the potential to contribute to the formulation of effective strategies to improve harm reduction implementation in the ED.

## Data Availability

In accordance with consent agreed upon with participants, and to protect the confidentiality of ED staff who work in a named research site, the authors will not be sharing their full data set. Data supporting the reported results are available throughout the manuscript text in the form of participant quotations.

## Abbreviations

ED: Emergency Department
AAN: Addiction Assessment Nurse
RAAC: Rapid Access Addiction Clinic
OPS: Overdose Prevention Site

## Footnotes

1 We acknowledge that OAT is both a long-term treatment for people who wish to abstain from unregulated opioid use, as well as a harm reduction intervention that offers overdose protection and withdrawal management while people continue to engage in use. For the purpose of this paper, we focus on OAT as a harm reduction intervention.

2 We acknowledge that the mandatory engagement of addiction specialists in the provision of OAT (with the exception of Suboxone®) is a specific practice of the study site, and that this may not be the case for other acute care settings.
